# Energy Intake Requirements in Pregnancy

**DOI:** 10.3390/nu11081812

**Published:** 2019-08-06

**Authors:** Jasper Most, Sheila Dervis, Francois Haman, Kristi B Adamo, Leanne M Redman

**Affiliations:** 1 Pennington Biomedical Research Center, Baton Rouge, LA 70808, USA; 2 School of Human Kinetics, University of Ottawa, Ottawa, ON K1N 6N5, Canada

**Keywords:** pregnancy, energy expenditure, energy intake, physical activity, metabolic rate, fetal development

## Abstract

Energy intake requirements in pregnancy match the demands of resting metabolism, physical activity, and tissue growth. Energy balance in pregnancy is, therefore, defined as energy intake equal to energy expenditure plus energy storage. A detailed understanding of these components and their changes throughout gestation can inform energy intake recommendations for minimizing the risk of poor pregnancy outcomes. Energy expenditure is the sum of resting and physical activity-related expenditure. Resting metabolic rate increases during pregnancy as a result of increased body mass, pregnancy-associated physiological changes, i.e., cardiac output, and the growing fetus. Physical activity is extremely variable between women and may change over the course of pregnancy. The requirement for energy storage depends on maternal pregravid body size. For optimal pregnancy outcomes, women with low body weight require more fat mass accumulation than women with obesity, who do not require to accumulate fat mass at all. Given the high energy density of fat mass, these differences affect energy intake requirements for a healthy pregnancy greatly. In contrast, the energy stored in fetal and placental tissues is comparable between all women and have small impact on energy requirements. Different prediction equations have been developed to quantify energy intake requirements and we provide a brief review of the strengths and weaknesses and discuss their application for healthy management of weight gain in pregnant women.

## 1. Introduction

Pregnancy is a determining period of future health for women and children. For the mother, poor pregnancy outcomes including excess gestational weight gain, gestational diabetes, hypertension and preeclampsia, or having a cesarean section increase the risk for future obesity, type 2 diabetes and cardiovascular diseases [1,2]. An infant born to a mother who experienced poor pregnancy outcomes is at increased risk for preterm birth, macrosomia and growth restriction, catch-up growth, adiposity and insulin resistance [3,4,5]. This increased risk for metabolic disease is not only limited to infancy or childhood, but is carried into adulthood [6,7].

One of the key factors for achieving optimal pregnancy outcomes is energy balance or the relationship between energy intake, energy expenditure and energy storage in maternal and fetal tissues. The energy intake requirement for a pregnant woman reflects the amount of energy needed to support maternal and fetal metabolism (energy expenditure) and fetal growth and accumulation of energy depots during pregnancy (energy storage). Individual energy requirements depend on numerous factors such as maternal pregravid body size, physical activity, and the physiological demands of pregnancy in each trimester. 

Between 1980 and 2010, various groups studied the energy requirements of pregnant women (Table 1). However, these studies come with limitations which are seldom considered when translating the findings into energy requirement recommendations. First, except for one study [8], the energy costs of weight gain are estimated based on all women studied. As such, women with excess weight gain likely cause an overestimation of the energy intake requirements because they deposited more energy than necessary, and their larger body size caused larger energy expenditure. Second, only one study estimated energy costs for recommended weight gain [8], but classification of both maternal pregravid body mass index (BMI) and recommendations for weight gain in pregnancy have since been revised. Third, women with obesity are significantly underrepresented. In 2014, the prevalence of obesity in women of childbearing age was 37% [9], whereas only three women with obesity have been enrolled in energy requirement studies in total [10]. Similarly, the prevalence of obesity is even larger among women of ethnic minorities (African American 57%, Hispanics 43%), yet the vast majority of women studied were Caucasian. These are important considerations for the assessment of energy requirements because both obesity and race have well-established effects on energy expenditure. The observation that offspring of African-American mothers are already prone to obesity suggests that energy metabolism may not only be differently regulated between races, but that this differential regulation is transmitted from one generation to the next, independent of environmental factors or lifestyle. Lastly, the main determinant of energy expenditure is body weight and thus, due to weight gain, energy expenditure increases during pregnancy. An accurate estimation of the energy costs that are a result of the increase in body weight as compared to the increased metabolic rate per body mass, or due to the fetus requires appropriate adjustment for the increase in weight gain. Herein, we review the energy intake requirements specific to the components of energy expenditure, including resting metabolic rate, fetal development, and physical activity. We also provide an estimate of the energy required to gain the appropriate amount of fat mass that relates to the recommended weight gain.

To assist women and healthcare providers in achieving healthy rates of gestational weight gain, prediction models have been developed to estimate energy intake requirements. In the final Section 5 of this review, we discuss the accuracy, strengths and limitations of these prediction models and propose future directions for studies that are needed to better understand the high prevalence of excess gestational weight gain among pregnant women.

## 2. Overview of Energy Intake Requirements of Healthy Pregnancy

### 2.1. Definition of Energy Requirements

Energy intake requirements are defined as dietary intake that is essential for an individual to sustain optimal health outcomes [16]. In a non-pregnant population, this is generally weight maintenance. In that case, the goal is to achieve long-term energy balance, by maintaining an energy intake that approximates energy expenditure. Total daily energy expenditure (TDEE) is the sum of energy expended to support the resting metabolic rate (RMR), diet-induced thermogenesis and physical activity. RMR accounts for 60–80% of TDEE and is dependent on body mass, sex, age and race. Diet-induced thermogenesis or the energy expended in response to the digestion and processing of food accounts for 10% of TDEE. Lastly, activity-related energy expenditure, the most variable component, includes energy expended during activity associated with daily living and structured bouts of exercise. The quality of the diet and type of activity may affect the determinants of energy balance, but these interactions are not discussed.

### 2.2. Energy Requirements in Pregnancy

Energy intake requirements in pregnancy are defined as dietary intake necessary to support optimal development of maternal tissues as well as to support fetal growth and development [1]. Therefore, requirements encompass energy intake that not only balances maternal and fetal energy expenditure but also provides additional energy for fetal growth, as well as the growth of maternal tissues such as fat mass, breast tissue, uterus and placenta. Thus, energy intake requirements in pregnancy are not aimed at weight maintenance, but for appropriate rates of weight gain, which in turn, minimizes the risks of adverse outcomes in the mother and her offspring.

For women of normal weight (body mass index 18.5 to 24.9 kg/m^2^), the recommended amount of weight gain during pregnancy is 11.5 to 16 kg, according to the 2009 Institute of Medicine guidelines [1]. Within this range of weight gain, the risk for non-elective cesarean delivery, postpartum weight retention, preterm birth, small- or large-for-gestational-age birth, and childhood obesity is minimized. The weight gain recommendations are inversely proportional to maternal body mass index at conception and, therefore, allow for more weight gain for women classified as underweight (12.5–18 kg) and less weight gain for women who are classified as overweight (7–11.5 kg) and with obesity (5–9 kg). For women with obesity, the weight gain recommendations have recently been challenged. Acknowledging limitations of available data [17,18,19], the Institute of Medicine was unable to suggest weight gain recommendations by obesity class [1]. Since then epidemiological studies propose that optimal pregnancy outcomes in women with severe obesity (body mass index ≥ 35 kg/m^2^) are achieved if weight gain is restricted to less than 5 kg or weight is maintained across pregnancy [20,21,22,23,24,25,26].

## 3. Energy Requirements for Expenditure

### 3.1. Total Energy Expenditure

#### 3.1.1. Appropriate Adjustment of Energy Expenditure Data

**Figure 1:**
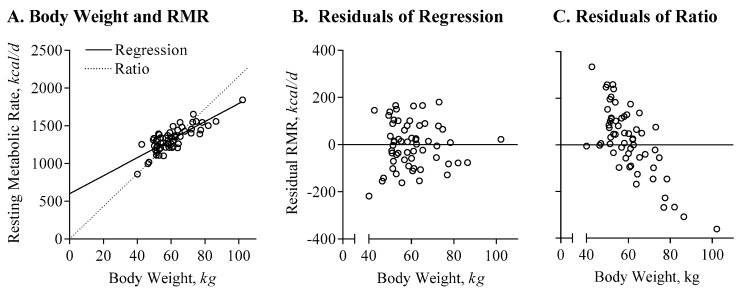
Appropriate adjustment of energy-expenditure data. (**A**) The association between body weight and resting metabolic rate is characterized by linear regression (solid line, RMR[*kcal/d*] = 598 + 11.97 × BW[*kg*], R^2^ = 0.66) and ratio (pointed line, RMR[*kcal/d*] = 21.56 × BW[*kg*], R^2^ = 0.22); of note, logarithmic or polynomial approximations do not improve the prediction of RMR (for both, R^2^ = 0.67). (**B**) Residual resting metabolic rate (RMR), calculated as measured RMR minus predicted RMR; based on the regression equation in (**A**); the residuals show no structural over- or underestimate based on body weight. (**C**) Residual RMR, based on the ratio equation; women with increased body weight have significant lower residual RMR, and would be considered as having a low adjusted metabolic rate.

As in non-pregnant populations, there is a linear association between energy expenditure and body size in pregnant women such that energy expenditure is higher in heavier women, see Figure 1 [10,27,28]. To be able to assess metabolic or behavioral differences or adaptations in energy expenditure between women, energy expenditure needs to be compared independent of body weight and this requires appropriate statistical adjustment of the raw (or absolute) data [27]. The consensus among energy metabolism experts is that the relationship between energy expenditure and body weight (or body composition) is best characterized by a linear regression with an intercept greater than 0 (EE = a + b × BW or EE = a + b × FFM + c × FM), and not by a simple ratio (EE = b × BW). A ratio assumes that the intercept of the relationship between energy expenditure and weight (or body composition) is equal to 0, see Figure 1A. Using a linear regression rather than a ratio to describe the relationship between body weight and energy expenditure reduces the coefficients ‘b’ for body weight (regression: 11.97, ratio: 21.56), improves the fit of the regression line (regression R^2^ = 0.66, ratio R^2^ = 0.22), and eliminates systematic bias for the estimation of individual variability, see Figure 1B,C. Using a ratio, women with lower body weight will be falsely characterized as having high energy expenditure (‘Residual’), and vice versa, women with more body weight will be falsely characterized as having low energy expenditure.

#### 3.1.2. Measurement by Doubly Labeled Water

Ideally, TDEE is objectively measured in free-living conditions by doubly labeled water [29]. The principle behind doubly labeled water is to indirectly assess carbon dioxide production from the differential dilution rates of deuterium (^2^H) and oxygen-18 isotope (^18^O) after a prescribed dose of the isotope-cocktail is ingested. After ingestion of the water, deuterium dilutes into the body water pool and is excreted from the body via urine, sweat and water vapor in breath. In contrast, the oxygen isotope is excreted not only through the loss of water but also as carbon dioxide. Thus, the difference in dilution rates of ^2^H and ^18^O is proportional to carbon dioxide production. Then, estimation of TDEE from carbon dioxide production requires an assumption or quantification of the oxygen consumption, which can be inferred by measurement or estimation of the respiratory quotient (EE[*kcal/d*] = VCO_2_ [*L/d*] × (1.1 + 3.9/RQ)) [29]. In most cases, the respiratory quotient or the ratio of carbon dioxide production to oxygen consumption will not be measured throughout the doubly labeled water period, and thus, requires assumptions. The best estimate for the respiratory quotient, which describes the macronutrient composition of the energy expenditure, is the food quotient, which describes the macronutrient composition of the energy intake. In most (pregnant and non-pregnant) studies, a respiratory quotient equivalent to a standard western-style diet of 0.86 is used (35–40% energy from fat) [30]. In 12 h overnight measurements of the energy expenditure, we observed no significant deviation from this commonly used value (unpublished).

The use of doubly labeled water to estimate TDEE is limited to a period of 7–14 days. Measurements of energy expenditure throughout pregnancy, therefore, require repeated, independent assessments of TDEE at the beginning and end of the assessment period of interest. The mean energy expenditure throughout pregnancy can then be calculated by the trapezoid method [31,32,33,34,35]. A limitation of this approach is the assumption of linearity between TDEE measurements. More frequent measurements of body weight and activity throughout this period, which is more feasible than TDEE, increase specificity, e.g., towards trimester-specific requirements. 

#### 3.1.3. First Trimester

**Figure 2:**
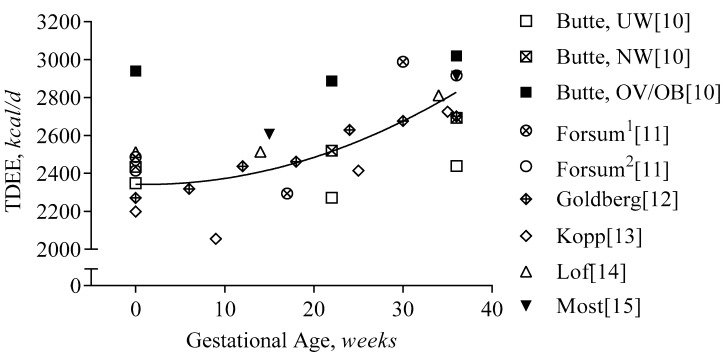
Total Daily Energy Expenditure during Gestation. Total daily energy expenditure (TDEE) is presented per study and gestational age (GA). The regression line represents the average increase during gestation (TDEE[*kcal/d*] = 2343 − 1 × GA[*weeks*] + 0.4 × GA^2^[*weeks*], R^2^ = 0.62). Forsum^1^ and Forsum^2^ indicate the two different cohorts in the study: both measured before pregnancy, but at different times during gestation; Butte reported the changes in TDEE per BMI class: ‘UW’ indicates underweight: BMI ≤ 19.8 kg/m^2^, ‘NW’ indicates normal weight: BMI 19.8–26 kg/m^2^, and ‘OV/OB’ indicates overweight/obesity: BMI ≥ 26 kg/m^2^.

Groups have performed cross-sectional comparisons of TDEE between pregnant and non-pregnant women [36,37,38,39]; however, these are outside of the scope of the current review. In Figure 2, we show published, time-series data of TDEE throughout pregnancy. On average, TDEE increases by approximately 15 kcal per day per week or by 420 kcal per day from pre-pregnancy to delivery. Prospective measurements of TDEE prior to pregnancy and in the first trimester have been reported in four studies of non-obese women [10,11,12,14]. One study included women with obesity (*n* = 3), but they were combined with overweight women [10]; another did not report changes in body weight during pregnancy [13]. On average, TDEE does not change during the first trimester, at an average increase in body weight of 3.2 kg [10,11,12,14]. Based on reported average changes in body mass and composition in these studies, we estimated the changes in TDEE after adjustment for changes in body mass. Using the linear regression equation of TDEE proposed by Gilmore and Butte et al. [31] that estimates TDEE from fat-free mass, fat mass and age (TDEE_pred_[*kcal/d*] = 1528 + 29.4 × FFM[*kg*] + 13.6 × FM[*kg*] − 21 × age[*years*]), body-weight-adjusted TDEE declines by 55 kcal/d from pre-pregnancy until 13 weeks of gestation across all studies. The components of TDEE that contribute to this decline could be of metabolic and/or behavioral origin, which we will discuss in the following sections.

#### 3.1.4. Second and Third Trimester

Only half a dozen studies have obtained prospective measurements of TDEE during the second and third trimester of pregnancy using doubly labeled water [10,11,12,13,14,15]. On average, TDEE increased by 18 kcal/d/week or ~420 kcal/d from week 13 to 36, at an average increase in body weight of 10.3 kg [10,11,12,13,14,15]. After adjusting for the change in body weight, the increase in TDEE was 170 kcal/d from early to late pregnancy (13 to 36 weeks). Using individual data from 112 women [10,15], we estimated that after adjustment for body weight change, the increase in TDEE was only ~75 kcal/d; 110 kcal/d in non-obese women and 45 kcal/d in women with obesity.

Based on these findings, energy expenditures from the second to the third trimester, are linearly related to weight gain with an estimated 45–170 kcal/d increase. Aside from metabolic and/or behavioral adaptations to pregnancy by the mother, the additional increase in TDEE also reflects the contribution of the fetus, since fetal metabolic rate has a disproportionately high energy expenditure to mass (100 kcal/d/kg body weight vs. ~25 kcal/d/kg in adults) [40,41]. The metabolic contribution of the placenta to TDEE is poorly understood but is likely an additional contributing factor. Therefore, to understand the individual determinants of energy requirements in pregnancy, it is important to assess the components of TDEE separately.

### 3.2. Basal Metabolism

#### 3.2.1. Measurement by Indirect Calorimetry

Differentiation of the metabolic and behavioral adaptations to pregnancy requires the assessment of individual components of TDEE, i.e., resting metabolic rate (RMR), diet-induced thermogenesis and activity-related energy expenditure. The largest component of TDEE is RMR, which accounts for 60–80% of daily energy requirements [10,11,12,14,28]. In majority of studies, RMR is measured by indirect calorimetry using a bedside ventilated hood system. In contrast to using doubly labeled water, oxygen consumption and carbon dioxide production are directly measured. The measurement of RMR occurs during fasting conditions over 30–60 min and extrapolated to 24 h. The RMR measurements have a high degree of accuracy, estimated at 3% [42], by test-retest reliability studies.

#### 3.2.2. First Trimester

**Figure 3:**
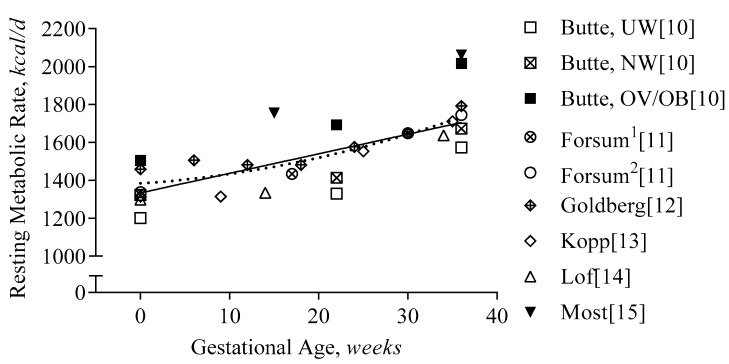
Resting Metabolic Rate during Gestation. Resting metabolic rate is presented per study and gestational age (GA). The regression line represents the average increase in the resting metabolic rate during gestation (RMR[*kcal/d*] = 1334 + 10.3 × GA[*weeks*], R^2^ = 0.55, using non-linear regression (pointed line) did not improve the prediction, R^2^ = 0.57). Forsum^1^ and Forsum^2^ indicate the two different cohorts in the study: both measured before pregnancy, but at different times during gestation; Butte reported the changes in RMR per BMI class: ‘UW’ indicates underweight: BMI ≤ 19.8 kg/m^2^, ‘NW’ indicates normal weight: BMI 19.8–26 kg/m^2^, and ‘OV/OB’ indicates overweight/obesity: BMI ≥ 26 kg/m^2^.

As with TDEE, only a few studies have assessed RMR before pregnancy and in early pregnancy. In Figure 3, we present data from studies with prospective assessments of RMR and TDEE. On average, RMR increased by 60 kcal/d during the first trimester (pre-pregnancy until week 13 [10,11,12,14] weeks gestation), and by 20 kcal/d after adjustment for the increase in body weight. A comparable increase in RMR (+30 kcal/d) was observed using individual data obtained by Butte and colleagues [10]. Thus, aside from changes in energy expenditure due to body weight, metabolic rate increases to a small extent, likely due to increased cardiac output [43] and/or hormonal changes, e.g., progesterone, human chorionic gonadotropin [44].

#### 3.2.3. Second and Third Trimester

During the second and third trimester, RMR increases by 390 kcal/d (or 17 kcal/d/week). Considering the change in body weight, the increase in RMR across studies was predicted at 170 kcal/d [31]. Thus >50% of the increase in RMR (215 kcal/d) is not explained by body weight but by the increased metabolic cost of pregnancy [10,11,12,14] that includes increased cardiac output [43], increased work of breathing [45], fetal activity, metabolic rate of fetal tissues [40].

### 3.3. Physical Activity/Exercise

Physical activity throughout pregnancy is crucial for healthy gestation, despite the increased physiological demands placed on the body [46,47,48]. Accordingly, many specialized guidelines and recommendations have been established [49,50,51,52,53] to promote women to accrue sufficient activity throughout gestation. These guidelines recommend at least 150 min of moderate physical activity every week. In support of these physical activity guidelines, meta-analyses [46,47,48] report that habitual exercise reduces the risks of excessive gestational weight gain by 32%, gestational diabetes by 38%, hypertension by 39%, moderate depression by 67% and of macrosomia by 39%. Despite the concomitant benefits of following physical activity guidelines during pregnancy, presently, less than 50% of women adhere to the guidelines [54,55,56,57,58].

#### 3.3.1. Measurement of Physical Activity

Physical activity can be quantified through measurement of the energy expenditure related to physical activity or through measurement of body movement. Using available calorimetry data, the easiest manner of quantifying activity-related energy expenditure is by subtracting metabolic rate and diet-induced thermogenesis (10% of TDEE) from total daily energy expenditure (activity-related EE = TDEE − 0.1x TDEE − RMR). Because body size not only affects RMR but also activity-related energy expenditure, the most widely used adjustment is determined by assessing the physical activity level (PAL), calculated as TDEE divided by RMR (PAL = TDEE/RMR). Of note, PAL may underestimate physical activity levels due to the increased RMR during pregnancy, but validation studies have yet to be performed. To assess the inter-individual variability within a cohort or over time, activity-related energy expenditure can also be assessed as the residual of the linear regression, TDEE = a + b × RMR; this value is positive for subjects with higher physical activity than average and is negative for subjects with lower physical activity than average independent of metabolic body size. Because residual activity-related energy expenditure is adjusted for the RMR, this value is directly proportional to the amount of physical activity. Calorimetric assessments of activity-related energy expenditure are limited by their relatively high costs and are, therefore, limited to small cohorts.

Devices that objectively measure physical activity such as accelerometers (i.e., Actigraph, Actical, RT3, FitBit) are widely used in scientific studies, and by the public to overcome the limitations associated with calorimetric measurements. Accelerometry assesses the magnitude and duration of movement and gravitational forces of activity to yield outcome variables such as time-stamped activity counts, duration and intensity. An advantageous feature that some accelerometers possess (e.g., Actical) is the inclusion of a pedometer or step counter, which are shown to be valuable objective tools for assessing physical activity interventions in pregnant women [59,60,61]. Accelerometers can be combined with heart rate monitors to obtain a more objective measure of intensity, relative to an individual’s fitness level [62,63]. To obtain a calorimetric estimate of physical activity (or total energy expenditure), accelerometry-variables are imputed into proprietary equations that predict, but do not measure, energy expenditure from body weight, height, and age of an individual.

Lastly, the easiest, cheapest and most established method for the evaluation of the level of physical activity include physical activity questionnaires, physical activity recall records and diaries. Specifically, during pregnancy, the ‘gold standard’ Physical Activity Questionnaire (PPAQ) is a widely accepted tool to assess the level of physical activity [64]. This self-administered questionnaire requires that participants report time spent in 32 different categories of activities including household/caregiving (13 activities), occupational (5 activities), sports/exercise (8 activities), transportation (3 activities), and in-activities (3 activities) [64]. Limitations of self-report methods include their low accuracy and reliability [65,66,67], as demonstrated by a significant overestimation of physical activity compared to accelerometers [65,68,69,70]. 

#### 3.3.2. First Trimester

**Figure 4:**
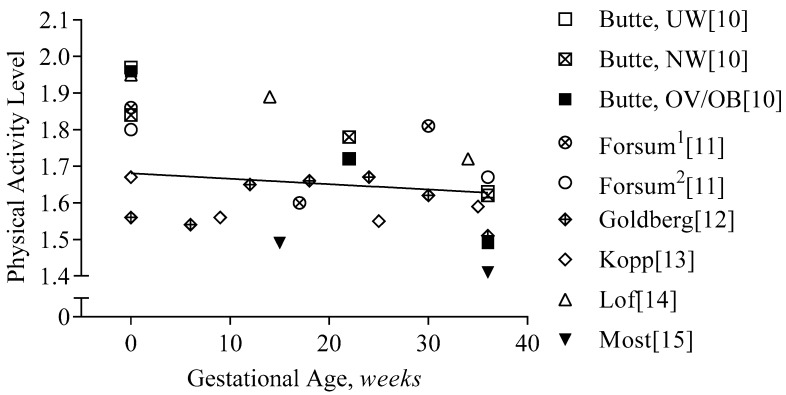
Physical activity level during gestation. Physical activity level is presented per study and gestational age (GA). The regression line represents the average decline in the physical activity level during gestation (PAL = 1.68 − 0.0015 × GA[*weeks*], R^2^ = 0.02). Forsum^1^ and Forsum^2^ indicate the two different cohorts in the study: both measured before pregnancy, but at different times during gestation; Butte reported the changes in PAL per BMI class: ‘UW’ indicates underweight: BMI ≤ 19.8 kg/m^2^, ‘NW’ indicates normal weight: BMI 19.8–26 kg/m^2^, and ‘OV/OB’ indicates overweight/obesity: BMI ≥ 26 kg/m^2^.

In Figure 4, we provide the physical activity level data of the calorimetry studies of pregnancy. During the first trimester, physical activity declined minimally in energy-requirement studies (−60 kcal/d, −2%, −0.08 PAL). Early in pregnancy, physical-activity-related energy expenditure accounted for 26–39% of energy expenditure in non-obese women [10,11,12,14] and for 23% in women with obesity [28].

#### 3.3.3. Second and Third Trimester

During the second and third trimesters, physical-activity-related energy expenditure decreased further by 3% (PAL, −0.12) [10,11,12,14]. Importantly, there is a significant variation between the studies; some report an increase in PAL of 0.37 [11], and others a decrease of −0.38 in women with overweight and obesity [10]; in a cohort of women who were exclusively obese (*n* = 54), PAL declined by 0.12 [15].

The variability in changes in activity is confirmed by studies using accelerometry. Some data show a decrease in physical activity level [55,71], while others reported no change [55,72,73]. The discrepancy in the literature may also relate to inconsistent methods of measurement, i.e., self-reported questionnaires vs. different various objective measures. Among a wide variety of demographic variables that may account for differences in physically active and inactive pregnant women, education and socio-economic status are consistently reported as predictors [54,74]. Therefore, while health care professionals should be encouraging all women to adopt an active lifestyle adherent to the recommendations of ≥150 min per week, they need to be cognizant of a woman’s demographic and socio-economic status and ensure their guidance is relevant and feasible.

## 4. Energy Cost for Weight Gain

### 4.1. Determination of Appropriate Weight Gain and Composition

In addition to the energy intake requirements needed to balance energy expenditure, total energy intake requirements also encompass energy deposition which during pregnancy includes maternal fat accumulation and fetal growth. The amount and composition of healthy weight gain are highly variable between women, particularly by BMI class [8]. Large epidemiological studies have assessed the range of gestational weight gain for each BMI class that are associated with the lowest risk for adverse pregnancy outcomes [17,18,19]. Based on these studies, the recommendations for appropriate weight gain are inversely related to pregravid BMI [1]. Translating these weight gain goals into energy intake requirements involves an understanding of weight gain composition because the components, i.e., fat mass and fat-free mass, have different contributions to the total weight gain and have different energy densities.

The differences in recommended weight gain have been attributed to the variance in fat mass accumulation, but not fat-free mass [8,15,75,76]. Thus, recommendations for higher weight gain, e.g., in underweight or normal weight as compared to overweight and obese women, imply that more fat mass is accumulated while the variability in fat-free mass is smaller, and from the perspective of energy content, less significant.

In most pregnancy studies, the density of fat-free mass is assumed to be 771 kcal/kg and the density of fat mass 9500 kcal/kg [10,31,34,35]. Thus, the determination of energy intake requirements for pregnancy is developed through the energy balance equation as:

EIR [*kcal/d*] = TDEE + (771[*kcal/kg*] × dFFM[*kg*] + 9500 [*kcal/kg*] × dFM[*kg*])

in which EIR is the estimated energy intake requirement, TDEE is the average total energy expenditure during pregnancy, and dFM and dFFM are the changes in fat and fat-free mass during pregnancy.

### 4.2. Measurement of Body Composition in Pregnancy

The measurement of changes in body composition during pregnancy is challenging (time-constraints) and costly, thus limited to small cohorts. Available methods include air displacement plethysmography, isotope dilution, skinfold thickness measurement, bioimpedance, and magnetic resonance imaging, which we have recently reviewed for their applications in pregnancy [77]. While all methods can be used in pregnancy, the trade-off between cost, time and precision determines the best choice. The most important consideration for the use of all methods in pregnancy is the adjustment for an increase in fat-free mass hydration during pregnancy. Based on classic data by van Raaij et al. [78], we have developed a prediction equation to estimate fat-free mass hydration dependent on gestational age that allows standardization of methods across studies:

Hydration FFM [*L/kg*] = 0.724 + 0.00008484 × GA[*weeks*] + 0.00001435 × GA[*weeks*]^2^

where FFM is fat-free mass and GA is gestational age; R^2^ = 0.998, *p* < 0.001, and

Density FFM [*kg/L*] = 1.1 − 0.00002988 × GA[*weeks*] − 0.00000731 × GA[*weeks*]^2^

where FFM is fat-free mass and GA is gestational age; R^2^ = 0.999, *p* < 0.001.

### 4.3. First Trimester

Independent of BMI class, the weight gain recommendations during the first trimester are 0.5 to 2.0 kg for all women [1]. To date, no study has assessed the composition of early pregnancy weight gain, specifically for women that adhered to this recommendation. Using a conservative estimate for the energy density of overall weight gain (7400 kcal/kg) [79], the energy requirements for weight gain in early pregnancy are ~3,700–14,800 kcal, or 40–165 kcal/d over 13 weeks.

### 4.4. Second and Third Trimester

The accumulation of fat-free mass during the third and second trimester is ~7–8 kg and comparable between women of different BMI classes [8,10,77]. Assuming an energy density of 771 kcal/kg fat-free mass, the energy required to support this fat-free mass accumulation is 5000–6000 kcal or 30–35 kcal per day, while studies report ~4,000–12,000 kcal [80,81] or 20–60 kcal/d. Considering the low energy density of fat-free mass, the variability in fat-free mass accumulation does not strongly impact energy requirements. In contrast, the variability in fat mass accumulation largely affects the energy requirements for weight gain in pregnancy.

Several studies have assessed the energy requirements for weight and fat mass gain during the second and third trimesters, yet few studies have determined these requirements specific to women who gained the recommended amount of weight. Based on Lederman et al. [8], pregnant women with a normal weight and recommended weight gain (BMI 19.8–26 kg/m^2^ and 11.5–16 kg, respectively) accumulated ~4 kg of fat mass between 14 and 37 weeks gestation. The energy intake requirements to support this energy deposition in fat is ~240 kcal/d over 22.7 weeks (i.e., 4 kg × 9500 kcal × kg^−1^/159 d = 240 kcal/d). For underweight or overweight women, gains in fat mass were 6.0 and 2.8 kg, and the respective energy intake requirements were 360 kcal/d and 165 kcal/d. For women with obesity, we have recently shown that recommended weight gain is achieved with an average 2.5 kg loss of fat mass (15 to 36 weeks of gestation) [15]. Therefore, instead of women with obesity depositing energy, 160 kcal/d of energy are mobilized from fat mass in the second and third trimester, and can, therefore, be subtracted from energy intake requirements. Given that 160 kcal/d is mobilized from fat tissue, while the accumulation of fat-free mass only requires 20–60 kcal/d, the factor of energy storage in the energy balance equation is negative and thus energy intake in pregnant women with obesity should be lower than energy expenditure.

## 5. Models that Estimate Energy Intake Requirements

Experimental reports evaluating energy intake requirements have been an essential tool to educate women and clinicians on the optimal energy intake during pregnancy. The Institute of Medicine, using available data at the time published ‘Equations to Estimate Energy Requirement for Pregnant Women’ (p. 316) [1]. The equation is based on the requirements of non-pregnant women (EIR = 354 − 6.91 × age [*years*] + PA × 9.36 × BW [*kg*] + 726 × height [*m*], in which EIR are estimated energy requirements, PA is physical activity (1.00 for PAL < 1.4, 1.12 for 1.4 ≤ PAL ≤ 1.59, 1.27 for 1.6 ≤ PAL ≤ 1.89, and 1.45 for 1.9 ≤ PAL ≤ 2.5), and BW is body weight. Importantly, by including physical activity as a variable, this model accounts for individual differences in the level and changes in activity. Energy costs for weight gain are assumed to be constant (180 kcal/d).

A dynamic model for the estimation of energy requirements for optimal weight gain has been developed to better acknowledge the different contributions of fat and fat-free mass in different BMI classes [34]. This model based on the most comprehensive studies of energy requirements in women without obesity [10,12,13], estimates the energy requirements to achieve the recommended rate for weight gain. The TDEE in this model is estimated using maternal age, body weight, and height, but not physical activity.

Importantly, both the Institute of Medicine and the model by Thomas and colleagues, have included all available data to develop an energy requirement prediction, and, therefore, have included data of women with excess and inadequate weight gain. Future models can, thus, be improved by restricting models to those women with appropriate weight gain only.

### 5.1. First Trimester

For the recommended weight gain of 0.5–2 kg in the first trimester [1], the Institute of Medicine and American College of Obstetricians and Gynecologists recommended women maintain pregravid energy intake throughout the first trimester because the energy costs for weight gain are considered minimal [1,82].

The energy intake requirement model suggests that all women should consume an additional 100–200 kcal/d to support first-trimester weight gain, assuming that physical activity does not decline during the first trimester [10,34]. For women with obesity, we have recently shown that the model significantly overestimates energy requirements by 400 kcal/d due to unaccounted lower levels of physical activity as compared to the non-obese cohorts, on which the model is based [28].

Similar to the Institute of Medicine, we estimate the energy requirements to be 50–150 kcal/d. An average decline in physical activity (PAL, −0.08 or −5%, or 60 kcal/d) may compensate those energy costs, but this compensation may differ greatly between individuals.

### 5.2. Second and Third Trimester

The Institute of Medicine estimates the energy requirements during the second and third trimester to be 340 kcal/d and 452 kcal/d, respectively [83]. The estimate is calculated as the sum of the increased energy expenditure over pregnancy (8 kcal/d/week) and the costs of energy deposition (180 kcal/d). The increase in energy expenditure is consistent with our estimates, whereas the proposed energy cost for deposition neglects BMI-specific weight gain recommendations. In 2016, the American College of Obstetricians and Gynecologists have, thus, adjusted their recommendations by acknowledging that ‘if you were overweight or obese, you may need fewer extra calories’ [82], without specific guidance.

The energy intake requirements model by Thomas et al. [34] estimated surplus energy requirements to be only 400–600 kcal/d for underweight and normal-weight women, and 220–350 kcal per day for overweight or obese pregnant women [34]. Because our estimates are based on the same data, they agree for underweight, normal-weight and overweight women. However, the model was developed from women without obesity or with obesity coupled with excess gestational weight gain, and, therefore, for women with obesity, the model overestimates fat mass gains and thus energy requirements [15].

During the second and third trimester, we estimate that the energy requirements for expenditure increase by 15 kcal/d per week, half of which is explained by the increase in body weight and half by increased metabolic rate largely due to cardiac output and fetal metabolism. The energy requirements for recommended weight gain differ by BMI class and range between 360 kcal/d for underweight women, to −165 kcal/d for women with obesity, with the variation explained by fat mass accumulation [8,77] at a constant fat-free mass accumulation equivalent of ~50 kcal/d.

## 6. Limitations and Practical Considerations

### 6.1. First Trimester

In clinical practice and research settings, it is challenging to quantify changes occurring in the first trimester. Such an assessment requires a measurement prior to pregnancy and, ideally, close to conception. In most studies, pre-pregnancy weights are estimated or abstracted from charts, which lacks the control required to define energy requirements accurately. Moreover, access to weight data only precludes interpretation of changes in body composition. Given the importance of the changes during the first trimester, innovative study designs need to be developed to overcome this barrier.

### 6.2. Variability in Energy Expenditure

Based on findings by Ravussin et al. in the late 1980s [84], studies have investigated whether variability in the RMR would increase the risk of increased fat accumulation. Indeed, multiple studies [27,85,86,87] support this hypothesis, showing that women with low RMR, that is residual RMR adjusted for body weight or fat-free mass, gained more weight than women with higher adjusted RMR. However, these studies adjusted energy expenditure using ratios, which prohibits them from reliable conclusions. Nevertheless, in a re-analysis of the data generated by Butte (1994), we confirmed the hypothesis of excess weight gain in women with lower adjusted RMR, who were not obese [27]; however, we did not observe this in women with obesity [15]. The origin of these differences in RMR remains to be determined since body mass and composition explain only ~70% of its variation [28].

One of the determinants of energy expenditure is race. As in non-pregnant cohorts [88], RMR in pregnant African-American women is lower than in Caucasian women (−80 kcal/d or −5%) [33]. This difference does not change over time and, therefore, adjusting energy intake recommendations early in pregnancy is likely sufficient to consider the lower energy requirements of African-American women during pregnancy.

We have reported that TDEE adjusted for body composition is ~600 kcal/d lower in women with obesity [28] as compared to women without obesity [10,27,31]. Obesity status itself, after adjustment for body weight, does not seem to affect RMR [28]. The difference in TDEE likely relates to differences in physical activity, the timing of energy expenditure measurements and ethnicity of the cohorts. Indeed, women with obesity were less active than women without obesity (PAL, 1.5 vs. 1.8) [28]; energy expenditure in the cohort with obesity was measured ~8 weeks earlier than in the cohort without obesity (8 weeks × 15 kcal/week = 120 kcal/d) [10,31,77]; and 50% of women in the obese cohort were African-American women, who have significantly lower energy expenditure (−8%) [33].

### 6.3. Communication of Recommendations

The communication of energy intake recommendations must be improved. Studies show that only one in four women feels appropriately informed on weight gain or energy intake goals suitable for pregnancy [89]. Aside from the notion that some form of communication has to be initiated, qualitative studies are needed to inform how such information should be best communicated to patients. For example, based on our recent study in women with obesity, the same energy intake target can be expressed differently; e.g., 2700 kcal/d, 17 kcal/d/kg body weight, the diet should be equivalent to early pregnancy energy expenditure, or eat ~5% less during pregnancy. Possible ways of communicating these results to patients (pregnant women) that may be received and perceived differently are ‘try and follow the energy intake requirement estimates using this equation/model’ [1,34], ‘try to maintain your usual diet throughout pregnancy’, or ‘try to eat 5% fewer calories than you are expending’. Importantly, such recommendations need to consider potential declines in physical activity. More work is needed to establish best practices for knowledge translation and dissemination of this information. How to best inform and guide women to make appropriate decisions and choices while pregnant is an active field of research.

## 7. Summary

In summary, energy intake requirements during the first trimester are minimally different from requirements before pregnancy (Table 2). Therefore, the Institute of Medicine model adopted from non-pregnancy provides valid estimates of energy requirements to match energy expenditure. During the second and third trimester, the development of the fetus and pregnancy-associated changes in the mother occur, i.e., increased blood volume, cardiac output, increase energy requirements by ~390 kcal/d, with small variations between women of differing body sizes. We estimate that 50% of this increase is proportional to the increase in body mass, and 50% is due to the increased metabolic costs of pregnancy and metabolic rate of the fetus. However, the energy requirements for weight gain differ significantly. We estimate that to achieve the recommendations for healthy gestational weight gain [83], underweight women store 360 kcal/d as fat tissue, normal-weight women store ~240 kcal/d, overweight women store 165 kcal/d, and women with obesity do not store energy at all, but instead mobilize 160 kcal/d from adipose tissue energy stores.

Current methods to assess energy intake requirements are limited in their prediction of energy expenditure and energy storage. Specifically, body composition and physical activity data during the first trimester are scarce. Given the importance of the first trimester for placental and fetal development [90], more studies are required to better inform patients and clinicians how to achieve healthier pregnancies. Particularly in women with obesity, energy expenditure (i.e., physical activity) and energy deposition are overestimated and thus would allow women to consume an amount of energy that would result in excess gestational weight gain. The level of physical activity is the most variable component of energy expenditure and requires consideration on an individual level. Commercially available accelerometers or built-in devices in mobile phones may assist women to maintain or increase their level of physical activity during pregnancy; currently, less than 50% of women meet the recommendations by leading institutions dedicated to promoting a healthy pregnancy. Adherence to recommendations related to energy intake and energy expenditure (physical activity) are key factors that will assist women in maintaining an appropriate energy balance thereby supporting the needs of pregnancy and the growing fetus.

## Figures and Tables

**Table 1:** Subject Characteristics of Cohorts in Energy Requirement Studies of Pregnancy.

First Author	Measurement Time Points	Cohort Size	Ethnicity	Age	BMI	Excess GWG
	Weeks Gestation	N	White, AA, Other			
Butte, UW [10]	0, 22, 36	17	15, 0, 2	31 ± 4	18.9 ± 0.8	18%
Butte, NW [10]	0, 22, 36	34	24, 4, 5	30 ± 3	22.1 ± 1.5	35%
Butte, OV/OB [10]	0, 22, 36	12	9, 2, 1	31 ± 5	28.8 ± 2.6	100%
Forsum^1^ [11]	0, 17, 30	22		29 ± 4	22.3 ± 3.1	
Forsum^2^ [11]	0, 36	19		28 ± 4	22.1 ± 3.4	
Goldberg [12]	0, 6, 12, 18, 24, 30, 36	12	12, 0, 0	29 ± 3	23.0 ± 3.3	
Kopp [13]	0, 9, 25, 35	10		29 ± 5	23.1 ± 2.1	10%
Lof [14]	0, 14, 34	23		30 ± 4	24.2 ± 4.8	
Most [15]	15, 36	54	28, 22, 4	28 ± 5	35.8 ± 5.0	67%

Age and BMI (body mass index) are presented as mean ± SD at enrollment. AA: African-American, Others: Hispanic, Asian, Biracial, ‘Other’, Excess GWG: gestational weight gain exceeding the guidelines at the time (1990), UW: underweight, NW: normal weight, OV: overweight, OB: obesity, Forsum^1^ and Forsum^2^ indicate the two different cohorts in the study: both measured before pregnancy, but at different times during gestation; Butte reported data per BMI class: ‘UW’ indicates underweight: BMI ≤ 19.8 kg/m^2^, ‘NW’ indicates normal weight: BMI 19.8–26 kg/m^2^, and ‘OV/OB’ indicates overweight/obesity: BMI ≥ 26 kg/m^2^.

**Table 2:** Summary of changes in body mass, composition and energy expenditure, proportional and independent of body weight changes.

	Tri- Mester	Body Weight	Fat-Free Mass	Fat Mass	TDEE	TDEE, Pred	TDEE, Res	RMR	RMR, Pred	RMR, Res
Non-Obesity										
Forsum1	1	2.1	0.2	1.9	−146	26	−172	73	25	48
Goldberg	1	1.7	0.2	1.5	181	22	159	26	21	5
Butte, UW	1	4.6	2.1	2.5	−45	91	−136	76	67	9
Butte, NW	1	3.4	1.7	1.7	51	68	−18	53	50	3
Butte, OV/OB	1	5.0	1.9	3.1	−31	93	−124	111	71	41
Lof	1	2.3	0.9	1.4	2	41	−39	33	32	1
Average	1	3.2	1.2	2.0	2	57	−55	62	44	18
Non-Obesity										
Forsum1	2 and 3	11.5	10.4	1.1	1226	312	914	381	199	181
Goldberg	2 and 3	8.0	5.9	2.0	252	193	59	298	129	168
Butte, UW	2 and 3	8.7	7.1	1.6	274	221	54	399	145	254
Butte, NW	2 and 3	11.7	8.9	2.8	284	290	−5	427	192	235
Butte, OV/OB	2 and 3	13.1	9.7	3.5	219	323	−104	531	215	316
Lof	2 and 3	10.8	7.5	3.3	341	256	85	349	173	176
Average		10.6	8.2	2.4	433	266	167	397	176	222
Obesity										
Most	2 and 3	8.5	8.2	0.3	335	237	98	335	150	185

Changes in kilograms and kcal per day. TDEE: Total daily energy expenditure, Pred: predicted, changes proportional to changes in body weight, Res: Residual, changes independent of changes in body weight, RMR: resting metabolic rate. Forsum1 indicate the two different cohorts in the study: both measured before pregnancy, but at different times during gestation; Butte reported the changes in PAL per BMI class: ‘UW’ indicates underweight: BMI ≤ 19.8 kg/m^2^, ‘NW’ indicates normal weight: BMI 19.8–26 kg/m^2^, and ‘OV/OB’ indicates overweight/obesity: BMI ≥ 26 kg/m^2^.
